# No increased risk of cancer death after endovascular aortic repair in a nationwide population-based cohort study

**DOI:** 10.1093/bjs/znag065

**Published:** 2026-06-03

**Authors:** Fredrik Lilja, Anders Wanhainen, Kevin Mani

**Affiliations:** Department of Surgical Sciences, Section of Vascular Surgery, Uppsala University, Uppsala, Sweden; Department of Surgical Sciences, Section of Vascular Surgery, Uppsala University, Uppsala, Sweden; Department of Diagnostics and Intervention, Surgery, Umeå University, Umeå, Sweden; Department of Surgical Sciences, Section of Vascular Surgery, Uppsala University, Uppsala, Sweden

## Abstract

**Introduction:**

The short-term benefits of endovascular aortic repair (EVAR) compared with open repair for the treatment of abdominal aortic aneurysm (AAA) patients are well established. However, concerns have been raised regarding a potential increased long-term cancer risk associated with EVAR, related to procedural and surveillance-related radiation exposure. The aim of this nationwide population-based cohort study was to evaluate whether EVAR is associated with an increased long-term cancer risk compared with open repair.

**Methods:**

All patients undergoing primary AAA repair for an intact AAA (ICD-10: I71.4) from January 2005 to February 2024 were identified from the Swedish National Patient Register. Previous and subsequent cancer diagnoses, as well as previous co-morbidities, for this cohort were recorded. Cause of death was retrieved from the Cause of Death Register. Inverse probability of treatment weighting (IPTW) was applied using propensity scores derived from baseline characteristics and co-morbidities. Weighted Cox regression models, with EVAR as the sole regressor, were then fitted for the event of a new cancer diagnosis and cancer related death.

**Results:**

Some 15 509 patients were identified (mean age of 73 years, 16.8% female, and 23.7% with a previous cancer diagnosis). After weighting, standardized mean differences for co-morbidities, age, sex, and recent hospital admissions were all within ±0.1. The median survival was 8.7 (95% c.i. 8.4 to 8.9) years for EVAR patients and 9.4 (95% c.i. 9.1 to 9.6) years for open repair patients. The median follow-up time was 4.9 (interquartile range (i.q.r.) 2.3 to 8.4) years for new cancer and 5.9 (i.q.r. 3.0 to 9.4) years for cancer-related death. Freedom from new cancer was lower in EVAR patients (HR 0.92 (95% c.i. 0.86 to 0.98)), whereas cancer-related survival was similar (HR 0.93 (95% c.i. 0.85 to 1.02)).

**Conclusion:**

EVAR was not associated with an increased risk of dying of cancer, but with an increased risk of being diagnosed with a new cancer. This should be interpreted carefully, as there is a clear risk of detection bias of otherwise unknown tumours due to routine imaging during EVAR surveillance.

## Teaser

Concerns have been raised that abdominal aortic aneurysm (AAA) patients treated with endovascular aortic repair (EVAR) are at higher risk of cancer development compared with patients treated with open repair. In this nationwide population-based cohort study, there was no increase in cancer-related mortality, but there was a small increase in the probability of getting a new cancer diagnosis, for EVAR patients.

## Introduction

The treatment of abdominal aortic aneurysm (AAA) patients has changed significantly since the introduction of endovascular aortic repair (EVAR)^1,2^. The majority of aortic repairs are now done with EVAR rather than open repair^3^. EVAR has the advantage of significantly lower perioperative mortality compared with open repair, but the survival curves converge over time. This convergence could be due to aneurysm-related mortality among EVAR patients^4^. Other potential causes, such as increased risk of cardiovascular death due to the untreated aneurysm sac or increased cancer-related mortality after EVAR, have been suggested^4–7^.

Patients treated with EVAR are inevitably exposed to ionizing radiation both due to fluoroscopy during the EVAR procedure and due to preoperative and postoperative CT^8^. Based on data from a cohort of survivors of the atomic bombings of Japan, in the Life Span Study, there is a linear relationship between radiation exposure and cancer development, although there is some controversy regarding whether there is a dose threshold below which the cancer risk is not increased^9^. It has been estimated that the risk of exposure-induced death after EVAR is about 0.8%^8^.

Indeed, the long-term follow-up of the EVAR-1 trial reported an increase in cancer mortality after 8 years of follow-up, although, overall, no such increase was observed^6^. Retrospective analyses suggest that EVAR increases the risk of abdominal cancer^5,10^. But there is a risk of residual confounders, selection bias, and detection bias in retrospective cohorts, therefore meriting further exploration in different national populations.

The aim of the present study was to test the hypothesis that there is increased risk of cancer development and cancer-related death in AAA patients treated with EVAR compared with patients treated with open repair.

## Methods

This was a nationwide retrospective cohort study using prospectively collected data from Swedish nationwide mandatory healthcare registers. All Swedish residents have a unique personal identification number that makes cross-referencing between these registers possible.

### Patients

All patients who were operated on because of an intact AAA from January 2005 to February 2024 were identified in the Swedish National Patient Register^11,12^, which contains data on all hospital admissions, from 1987, and outpatient appointments with specialized care, from 2001. These patients were selected based on having a combination of an ICD-10^13^ code for an intact AAA (I71.4) and a Nordic Medico-Statistical Committee (NOMESCO) code for AAA repair (*supplementary material S1*). When a patient had NOMESCO codes for both EVAR and open repair, the patient was classified as being treated with EVAR. Any previous AAA surgery was identified in the National Patient Register dating back to 1998. All patients with a previous AAA repair were excluded from the analysis. Data on co-morbidities were retrieved from the National Patient Register dating back to 1998. Time and cause of death were retrieved from the Swedish Cause of Death Register. Patients treated with EVAR were compared with patients treated with open repair.

### Outcome

The two primary outcomes were new cancer diagnosis and cancer-related death. The former was defined as an ICD-10 diagnosis of any cancer after AAA repair that was not present before the operation. The latter was defined as having at least one cancer diagnosis listed among the causes of death in the Cause of Death Register. Secondary outcomes were intestinal cancer (C15–C26), cancer in respiratory organs (C30–C39), cancer in urinary organs (C64–C68), and cancer in haematopoietic tissue (C81–C96).

### Statistical analysis

**Figure 1:**
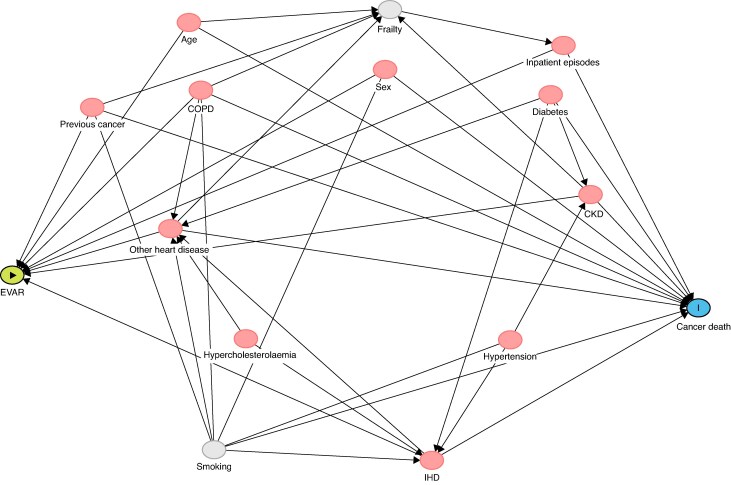
Directed acyclic graph showing possible biasing paths between exposure and outcome Pink represents observed variables and grey represents unobserved variables. EVAR is the exposure and cancer death is the outcome. EVAR, endovascular aortic repair; COPD, chronic obstructive pulmonary disease; IHD, ischaemic heart disease; CKD, chronic kidney disease.

To adjust for confounders, inverse probability of treatment weights were used. These weights were calculated from a propensity score created on the basis of a logistic regression with EVAR as the dependent variable utilizing Python package statsmodels^14^ (*supplementary material S2*). The choice of independent variables for this regression was based on a directed acyclic graph (DAG) based on prior knowledge (*Fig. 1*). The ability of the propensity score to predict treatment was evaluated using a receiver operating characteristic (ROC) curve, yielding an area under the curve (AUC) of 0.7 (*supplementary material S5*). The inverse probability of treatment weights were standardized and truncated at the 1st and 99th percentiles to avoid extreme weights (*supplementary material S3 and S4*)^15^.

Survival was assessed using weighted Kaplan–Meier curves. HRs were calculated with Cox regression, using EVAR as the sole regressor and the inverse probability of treatment weights as weights using Lin-Wei estimation to compute robust errors^16^. The Python package used for Kaplan–Meier and Cox regression was lifelines^17^.

A sensitivity analysis excluding all patients with a reported cancer diagnosis before AAA repair was performed.

Follow-up times are reported as median (interquartile range (i.q.r.)).

### Ethical approval

The study was approved by the national ethical review board (DNR 2014/078).

## Results

**Figure 2:**
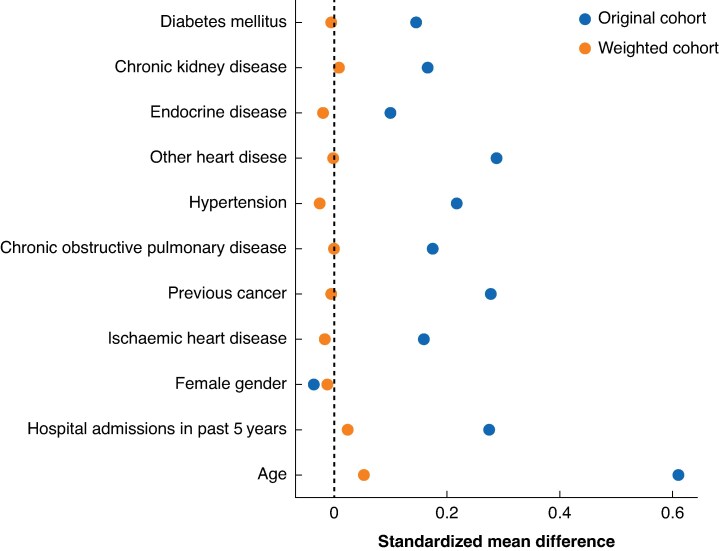
Love plot showing covariate balance before and after inverse probability of treatment weighting (IPTW)

Some 15 509 patients undergoing primary aortic repair of in intact AAA were identified, of whom 60.1% were treated with EVAR (*Table 1*). The covariate balance between groups after weighting is shown in *Fig. 2*.

**Table 1:** Baseline characteristics and co-morbidities for the patients in the unmatched cohort

	Open repair	EVAR
Age (years), mean(s.d.)	70.5(7.0)	74.8(6.9)
Hospital admissions in past 5 years (*n*), median (i.q.r.)	1 (0 to 2)	1 (0 to 3)
Female	17.6	16.2
Ischaemic heart disease	31.6	39.2
Previous cancer	16.8	28.3
Chronic obstructive pulmonary disease	12.4	18.7
Hypertension	49.0	59.8
Other heart disease	22.5	35.4
Endocrine disease (including hyperlipidaemia)	25.9	30.4
Chronic kidney disease	3.5	7.2
Diabetes mellitus	10.7	15.6

Values are % unless otherwise indicated. EVAR, endovascular aortic repair.

The median survival was 8.7 (95% c.i. 8.4 to 8.9) years for EVAR patients and 9.4 (95% c.i. 9.1 to 9.6) years for open repair patients. The median follow-up time was 4.9 (i.q.r. 2.3 to 8.4) years for new cancer and 5.9 (i.q.r. 3.0 to 9.4) years for cancer-related death.

**Figure 3:**
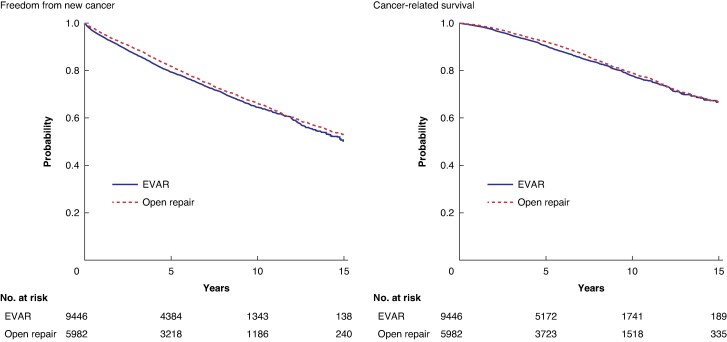
Long-term cancer development and cancer related mortality based on open or endovascular repair **a** Freedom from new cancer diagnosis after AAA repair. **b** Cancer-related survival after AAA repair. AAA, abdominal aortic aneurysm; EVAR, endovascular aortic repair.

Kaplan–Meier estimates for freedom from new cancer and for cancer-related survival and the Cox regression analysis revealed that freedom from new cancer was lower in EVAR patients (HR 0.92 (95% c.i. 0.86 to 0.98)), whereas cancer-related survival was similar (HR 0.93 (95% c.i. 0.85 to 1.02)) (*Fig. 3*).

Among the secondary endpoints, there was no difference in rate of diagnosed intestinal cancer (HR 0.97 (95% c.i. 0.83 to 1.13)), cancer in respiratory organs (HR 1.02 (95% c.i. 0.88 to 1.19)), or cancer in urinary organs (HR 1.08 (95% c.i. 0.89 to 1.30)). However, cancer in haematopoietic tissue (HR 1.37 (95% c.i. 1.04 to 1.81)) was statistically more prevalent in the EVAR group.

After excluding patients with a cancer diagnosis before AAA repair (8440 patients) in the sensitivity analysis, there was no significant difference for freedom from cancer (HR 0.93 (95% c.i. 0.87 to 1.02)) or freedom from cancer-related death (HR 0.93 (95% c.i. 0.83 to 1.05)).

## Discussion

In this nationwide population-based study, no statistically significant difference in cancer-related mortality was observed between EVAR and open repair, despite a slightly higher incidence of cancer diagnosis during follow-up after EVAR. These findings should, however, be interpreted with caution because the increased imaging surveillance after EVAR may have introduced detection bias through more frequent CT examinations, and because the observational design precludes any firm conclusions regarding causality.

Radiation-induced cancer development is a slow process^18^ and, even when malignancy develops, it usually takes time before it can lead to death. As the mean age for AAA repair in this cohort is 73 years and the median survival after aortic repair is 8.7 and 9.4 years for EVAR and open repair respectively and the more common cause of death is cardiovascular events^6^, there is, for most patients, simply not enough time to develop and die from a radiation-induced cancer. Furthermore, the follow-up after EVAR is in many cases done with repeated CT^19^. These are likely to find tumours that may be clinically insignificant due to advanced age and co-morbidities, which would explain the discrepancy between increased cancer incidence without a corresponding increase in cancer-related mortality. In the present IPTW cohort, the difference in new cancer diagnosis was most visible after 3–5 years with a higher rate of new cancer diagnoses in the EVAR group (*Fig. 3*). This relatively short time span between EVAR and cancer diagnosis indicates that this may be an effect of detection bias. On the other hand, cancer in haematopoietic tissues, which was more common among EVAR patients, is known to have a shorter latency interval^20^.

The underlying assumption for the hypothesis of causality between EVAR and cancer is that ionizing radiation, from CT and fluoroscopy, causes damage to DNA and in that way increases the likelihood of tumorigenesis. This belief is supported by data from the Life Span Study that estimated the radiation dose for survivors of the atomic bombings and recorded whether they developed cancer. It found a clear dose–response relationship for high radiation doses. For the purpose of radiation protection, this has been extrapolated to low doses, the so-called linear no-threshold (LNT) model. It is, however, unclear what the cancer risk is after exposure to low doses of radiation, and the LNT model has, indeed, been questioned^9,21^.

The literature on cancer risk after EVAR is scarce, but points to increased cancer risk. There are several studies in which the radiation dose during EVAR and follow-up CT has been recorded and various algorithms, based on the LNT model, have been applied to calculate cancer risk, which was found to be non-negligible^22–24^. In the 15-year follow-up of the EVAR-1 trial, there was no overall increased risk of cancer death, when analysed according to intention to treat^6^. After 8 years, an increased risk of cancer mortality was observed^6^. No per protocol analysis of cancer mortality was reported^6^. A population-based study from England with 14 150 patients showed an increased risk of development of abdominal cancer and a decrease in cancer-free survival for AAA patients treated with EVAR^5^. This study adjusted for centres using CT as the follow-up method *versus* centres that did not, reducing the presumed effect of detection bias. A retrospective study of 18 802 AAA patients registered with a German insurance company found no difference in overall cancer incidence between EVAR and open repair patients, but an increased incidence of abdominal cancer in EVAR patients^10^. A meta-analysis of these two studies addressing the incidence of abdominal cancer after AAA repair concluded that the HR for abdominal cancer was significantly higher for EVAR patients, while the cumulative incidence after 7 years was not^25^.

The major strengths of this study are the nationwide coverage and the long follow-up. All patients using Swedish healthcare and who die in Sweden will be included in follow-up. In Sweden, any contact with specialized care during follow-up will be recorded together with the diagnosis that caused the contact. Hence, data on new cancer diagnoses are deemed reliable. The only way to be lost to follow-up is to move abroad. For the age group of the patients in the present study, the rate of international migration is likely very small. Moreover, data on co-morbidities are collected from registers and are not self-reported by patients. The Swedish Cause of Death Register provides near-complete national coverage, as it includes all deaths reported to the tax authorities. An underlying cause of death is documented in over 99% of cases. The accuracy of these data is considered high, particularly for deaths due to malignant neoplasms^26^.

As in all non-randomized studies, there is a risk of confounding. In this study, the complexity of aneurysm anatomy is likely a variable affecting the choice of method that could not be accounted for. Smoking is another, but is likely, in part, to be accounted for by other variables with a large covariance with smoking, such as ischaemic heart disease and chronic obstructive pulmonary disease. Although not a confounder, the number of times EVAR patients undergo CT during follow-up might introduce detection bias, as clinically insignificant tumours may be detected at a higher rate in EVAR patients compared with open repair patients.

## Supplementary Material

znag065_Supplementary_Data

## Data Availability

Data related to this study are not publicly available due to restrictions related to patient privacy and ethical approvals but are available from the corresponding author upon reasonable request and with relevant permissions.
