# Supplementary material for: No increased risk of cancer death after endovascular aortic repair in a nationwide population-based cohort study
Source: Br J Surg. 2026 Jun 3;113(7):znag065. doi: 10.1093/bjs/znag065 (PMC13327106; doi:10.1093/bjs/znag065)
Supplement: znag065_Supplementary_Data [file znag065_supplementary_data.docx]

No Increased risk of Cancer Death after Endovascular Aortic Repair in a nationwide population-based cohort study

Fredrik Lilja^1^, MD, Anders Wanhainen^1,2^, MD PhD, Kevin Mani^1^, MD PhD

^1^Department of Surgical Sciences, Vascular Surgery, Uppsala University, Uppsala, Sweden

^2^ Department of Perioperative and Surgical Sciences, Surgery, Umeå University, Umeå, Sweden

**Corresponding author.** Dr Fredrik Lilja, Department of Surgical Sciences, Section of Vascular Surgery, Uppsala University, 75185 Uppsala, Sweden. ORCID-ID: 0009-0005-6968-9189

**Supplementary Materials - Index**

| **Supplementary Methods** |  |
| --- | --- |
| Definitions, S1  Propensity score regression, S2  Construction of weights, S3 | *page 2*  *page 3*  *page 3* |
| **Supplementary Figures and Tables** |  |
| Distribution of propensity scores and inverse probability treatment weights, S4  Receiver operator characteristics (ROC) curve, S5 | *page 4*  *page 5* |
|  |  |

**Supplementary Methods**

**S1**, definitions

Patients were included in the initial cohort if they had a registered admission in the Swedish Inpatient Register between 2005-01-01 and 2023-12-31 with ICD code I71.4, intact abdominal aortic aneurysm as their main diagnosis and a NOMESCO code suggesting open or endovascular aortic repair.

The NOMESCO codes PDG10, PDG20, PDG21, PDG22, PDG23, PDG24, PDG30, PDG35, PDG99, VBJ3, VBJ35 and VBJ23 were considered open aortic repair procedures and PDQ10, PDQ21, VBQ35 and VBQ34 were considered endovascular aortic repair procedures. All re-do procedure were excluded based on data from the Swedish Inpatient Registry dating back to 1998.

For the selected cohort data from the Swedish Inpatient and Outpatient Registers ranging from January 1997 to December 2023 were collected regarding comorbidities using ICD codes I20-I25 for ischemic heart disease (IHD), I30-I52 for non-ischemic heart disease, I10-I15 for hypertension, I60-I69 for cerebrovascular disease (CVD), E10-E14 for diabetes mellitus (DM), J40-J47 for chronic obstructive pulmonary disease (COPD), E70-E90 for endocrine disease (including hypercholesterolemia) and N17-N19 for chronic kidney disease (CKD).

The following were considered cancer diagnoses: C00-C14, C15-C26, C30-C39, C40-C41, C43-C44, C45-C49, C50-C50, C51-C58, C60-C63, C64-C68, C69-C72, C73-C75, C76-C80, C81-C96 and C97-C97.

**S2**, regression for propensity score

Logit Regression Results

==============================================================================

Dep. Variable: EVAR No. Observations: 15509

Model: Logit Df Residuals: 15498

Method: MLE Df Model: 10

Date: Thu, 05 Feb 2026 Pseudo R-squ.: 0.09014

Time: 22:27:18 Log-Likelihood: -9488.8

converged: True LL-Null: -10429.

Covariance Type: nonrobust LLR p-value: 0.000

=====================================================================================

coef std err z P>|z| [0.025 0.975]

-------------------------------------------------------------------------------------

age_std 0.5815 0.019 30.613 0.000 0.544 0.619

female -0.2079 0.045 -4.584 0.000 -0.297 -0.119

prevIHD 0.0764 0.039 1.948 0.051 -0.000 0.153

prevCA 0.4429 0.042 10.670 0.000 0.362 0.524

prevCOPD 0.3618 0.049 7.331 0.000 0.265 0.458

prevHT 0.2289 0.035 6.536 0.000 0.160 0.298

prevHD 0.2909 0.041 7.039 0.000 0.210 0.372

prevED -0.0036 0.044 -0.083 0.934 -0.089 0.082

prevCKD 0.4141 0.086 4.810 0.000 0.245 0.583

prevDM 0.3196 0.054 5.898 0.000 0.213 0.426

n_epidodes_5y_std 0.1327 0.020 6.713 0.000 0.094 0.171

=====================================================================================

**S3,** Construction of weights

Definition of the inverse probability of treatment weights (IPTW)

$$\boldsymbol{w}_{\boldsymbol{i}}^{\boldsymbol{IPTW}}\boldsymbol{=}\left\{ \begin{aligned} \frac{\boldsymbol{P}\boldsymbol{(}\boldsymbol{A}\boldsymbol{=}\boldsymbol{1}\boldsymbol{)}}{\boldsymbol{P}\boldsymbol{(}\boldsymbol{A}\boldsymbol{=}\boldsymbol{1}\boldsymbol{|}\boldsymbol{X}_{\boldsymbol{i}}\boldsymbol{)}}\boldsymbol{,}\boldsymbol{if} \boldsymbol{A}_{\boldsymbol{i}}\boldsymbol{=}\boldsymbol{1} \\ \frac{\boldsymbol{P}\boldsymbol{(}\boldsymbol{A}\boldsymbol{=}\boldsymbol{0}\boldsymbol{)}}{\boldsymbol{1}\boldsymbol{-}\boldsymbol{P}\boldsymbol{(}\boldsymbol{A}\boldsymbol{=}\boldsymbol{1}\boldsymbol{|}\boldsymbol{X}_{\boldsymbol{i}}\boldsymbol{)}}\boldsymbol{,}\boldsymbol{if} \boldsymbol{A}_{\boldsymbol{i}}\boldsymbol{=}\boldsymbol{0} \end{aligned} \right.$$

Where:

- A = 1 denotes EVAR and A = 0 denotes open repair.
- X denotes the covariates used to create the propensity score.
- Hence, P(A=1 | X_i_) is the propensity score.

Next the IPTW are truncated.

$$\boldsymbol{w}_{\boldsymbol{i}}^{\boldsymbol{trunc}}\boldsymbol{=}\left\{ \begin{aligned} \boldsymbol{q}_{\boldsymbol{0}\boldsymbol{.}\boldsymbol{01}}\boldsymbol{,}\boldsymbol{if} \boldsymbol{w}_{\boldsymbol{i}}^{\boldsymbol{IPTW}}\boldsymbol{<}\boldsymbol{q}_{\boldsymbol{0}\boldsymbol{.}\boldsymbol{01}} \\ \boldsymbol{w}_{\boldsymbol{i}}^{\boldsymbol{IPTW}}\boldsymbol{,}\boldsymbol{if} {\boldsymbol{q}_{\boldsymbol{0}\boldsymbol{.}\boldsymbol{01}}\boldsymbol{\leq}\boldsymbol{w}}_{\boldsymbol{i}}^{\boldsymbol{IPTW}}\boldsymbol{\leq}\boldsymbol{q}_{\boldsymbol{0}\boldsymbol{.}\boldsymbol{99}} \\ \boldsymbol{q}_{\boldsymbol{0}\boldsymbol{.}\boldsymbol{99}}\boldsymbol{,}\boldsymbol{if} \boldsymbol{w}_{\boldsymbol{i}}^{\boldsymbol{IPTW}}\boldsymbol{>}\boldsymbol{q}_{\boldsymbol{0}\boldsymbol{.}\boldsymbol{99}} \end{aligned} \right.$$

Where:

- q_0.01_ is the 1^st^ percentile of w^IPTW^
- q_0.99_ is the 99^th^ percentile of w^IPTW^

**Supplementary Figures and Tables**

**S4.** Distribution of propensity scores and inverse probability treatment weights (IPTW), EVAR, endovascular aortic repair.

**
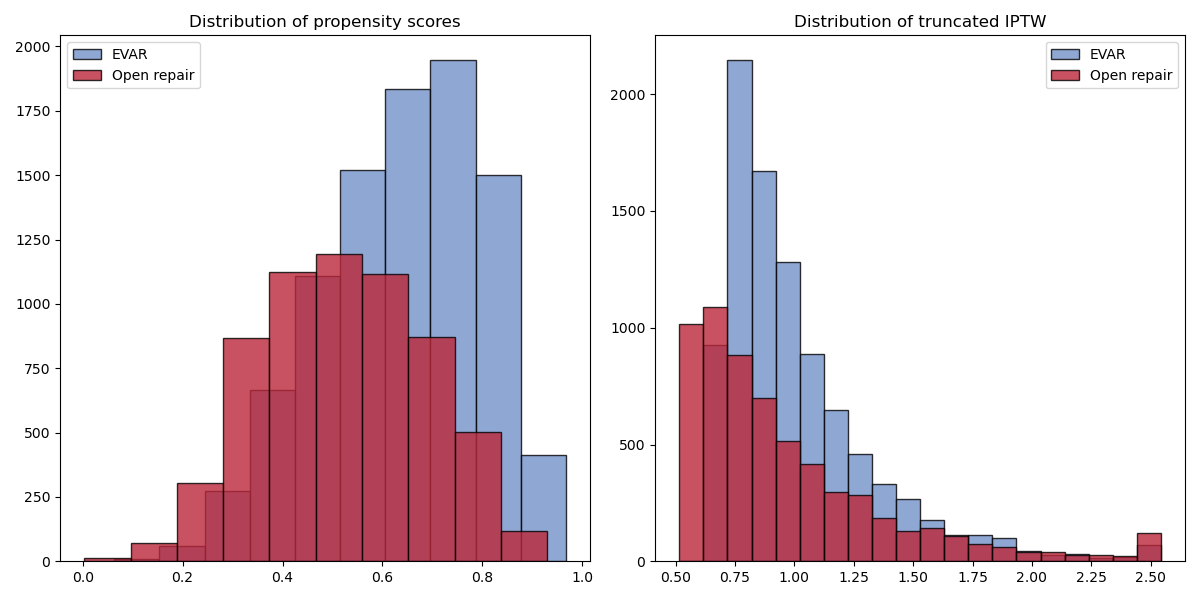
**

**S5.** Receiver operator characteristics (ROC) curve describing the ability of the propensity score to predict endovascular aortic repair.

**
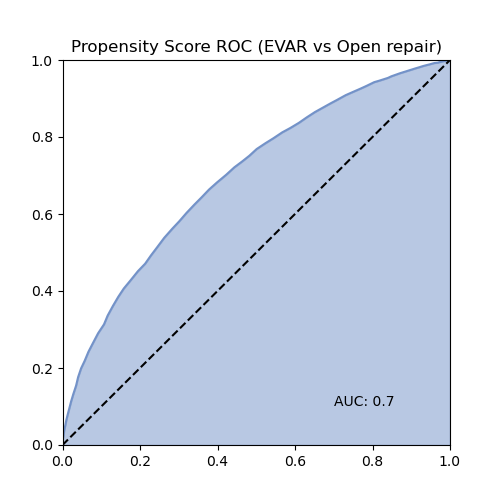
**
